# Innate Immune Response Against Batai Virus, Bunyamwera Virus, and Their Reassortants

**DOI:** 10.3390/v16121833

**Published:** 2024-11-26

**Authors:** David D. J. A. Zöller, Josefin Säurich, Julia Metzger, Klaus Jung, Bernd Lepenies, Stefanie C. Becker

**Affiliations:** 1 Institute for Parasitology, University of Veterinary Medicine Hannover, 30559 Hannover, Germany; david.zoeller@tiho-hannover.de; 2 Research Center for Emerging Infections and Zoonoses, University of Veterinary Medicine Hannover, 30559 Hannover, Germany; bernd.lepenies@tiho-hannover.de; 3 Institute for Animal Genomics, University of Veterinary Medicine Hannover, 30559 Hannover, Germany; josefin.saeurich@t-online.de (J.S.); julia.metzger@tiho-hannover.de (J.M.); klaus.jung@tiho-hannover.de (K.J.); 4 Institute for Immunology, University of Veterinary Medicine Hannover, 30559 Hannover, Germany

**Keywords:** Elliovirales, Peribunyaviridae, emerging infectious disease, antiviral response, interferon

## Abstract

*Orthobunyaviruses* (OBVs) represent a diverse group of RNA viruses, encompassing a progressively increasing number of arboviruses that cause disease in both humans and livestock. Yet, studies investigating these viruses remain scarce despite the critical importance of such knowledge for assessing their zoonotic potential. In this study, we conducted an evaluation of the early immune response against the understudied Batai virus (BATV), as well as the influence of reassortment with the Bunyamwera virus (BUNV) on this response. Using RNA sequencing of infected murine bone marrow-derived dendritic cells, complemented by qPCR assays, we assessed the innate immune response at the transcriptome level. Additionally, we extended the qPCR analysis by including human THP-1-derived dendritic cells and ovine SFT-R cells to identify differences across species. Our results provide the first evidence that BATV elicits a strong innate immune response compared to BUNV, which largely evades early detection. Reassortants exhibited intermediate phenotypes, although unique changes in the early immune response were found as well. These findings provide a starting point for a better understanding of the immune response to BATV. Furthermore, they raise the question of whether reassortment induces changes in the innate immune response that might contribute to the differences in pathogenicity between reassortant OBVs and their parental generations.

## 1. Introduction

*Orthobunyaviruses* (OBVs) constitute one of the largest and most diverse genera of RNA viruses, predominantly known for their impact on the health of humans and livestock, especially ruminants [1,2,3]. Considering the increasing global risk posed by arboviruses and the fact that many OBVs traverse the world as unwelcome passengers within hematophagous arthropods, this health threat becomes even more pronounced [1,4,5]. Mirrored is this increasing risk on the one hand by the serious diseases induced by OBVs like La Crosse virus (LACV) [6], Oropouche virus [7,8], Cache Valley virus (CVV) [9], and Schmallenberg virus (SBV) [10], and, on the other hand, by the continuous emergence of new, potentially pathogenic, OBVs [11,12,13,14,15,16,17]. This frequent emergence of new OBVs can be, for the greater part, attributed to their typical bunyavirus genome structure, which consists of three negative-sense, single-stranded RNA segments. Such a segmentation enables, under co-infection of host cells with compatible bunyaviruses, the generation of reassortant viruses with new segment compositions [3,18,19]. Such processes drive enormous genetic and biological diversity, eventually conferring significant fitness advantages or disadvantages [20]. The effect such reassortment events can have on the pathogenicity of viruses and, consequently, on the impact within a One Health context has already been described multiple times. Prominent examples are the Iquitos virus [16,21], SBV [10,13,18,22], and Ngari virus (NRIV) [23,24,25]. From a human perspective, the most alarming of these reassortants is arguably NRIV due to its capacity to cause fatal haemorrhagic fevers in both humans and ruminants [25]. What makes this case particularly fascinating is that, although NRIV is associated with a severe phenotype, the parental viruses, Bunyamwera (BUNV) and Batai (BATV), cause much milder disease manifestations. BUNV infections can lead to reproductive complications and malformations in ruminants, mild symptoms including fever, rashes, and myalgia in humans, and the occasional central nervous system infection in humans and horses [25,26]. In contrast, there are no described clinical symptoms in ruminants due to BATV infections yet [27,28], although, for the ‘Chittoor’ variant, mild illness is reported. Regarding human infections, influenza-like symptoms are recorded in the context of BATV infections [25,29]. Additionally, some evidence for the occurrence of BATV-associated encephalitis in harbour seals is present [30]. The basis of the increased pathogenicity of NRIV remains elusive, largely due to a general lack of studies regarding OBVs, their reassortment, and their interaction with the host’s immune system. This issue is further intensified by the fact that the majority of the conducted research is focused only on a small subset of OBVs [2,3]. As a prime example, the interplay between BUNV and the host’s immune response is comparatively well-characterised, particularly the influence of BUNV upon the type I interferon (IFN-I) response [31,32,33,34,35,36,37,38,39,40,41,42,43], while studies on the immune response to BATV are limited to a few serological insights [27,28,44,45,46]. Information regarding the reassortant NRIV is entirely missing.

The absence of such insights into host innate immunity-mediated restrictions and viral evasion mechanisms has wide-reaching negative effects, not least on the development of antivirals and vaccines [38]. This is a significant problem considering that OBVs, like these three, may be agents of serious public health threats and urgently need further research [2,29,47,48].

To address this gap, we designed a project aimed at extending the limited knowledge of how mammalian cells respond to infections with understudied OBVs. We aimed to gain insights into the initial immune response against BATV and BUNV, as well as the influence of reassortment on this response. Therefore, we used the experimentally generated reassortants Ngari-like virus (lNRIV), which shares the same genomic organisation as NRIV but harbours the M-segment of a German BATV isolate, and Batunya virus (BAYAV), which currently has no natural counterpart [49]. Utilising RNA sequencing, we investigated both the fundamental immune response and virus-specific differences. Subsequently, seven representative differentially expressed genes were used for an extended assessment of the differences between OBVs, including ovine, murine, and human cells, to evaluate species variance. This study provides the first indications for the immunogenicity of BATV, as well as reassortment-induced changes in the extent of the initial immune response.

## 2. Materials and Methods

### 2.1. Cell Culture

BHK-21 cells (*Mesocricetus auratus*; CCVL L 0179) were cultured in MEM with Earle’s salts (MEM-E) (Capricorn Scientific, Ebsdorfergrund, Germany) supplemented with 2 mM stable L-glutamine (Capricorn Scientific, Ebsdorfergrund, Germany), 1% penicillin–streptomycin (100 units/mL) (PAN-Biotech GmbH, Aidenbach, Germany), and 10% fetal bovine serum (FBS) (Capricorn Scientific, Ebsdorfergrund, Germany) at 37 °C in 5% CO_2_. Culturing of SFT-R cells (Ovis aries; CCVL 0043) happened under the same conditions with the addition of 0.05 mM 2-Mercaptoethanol (Gibco, Paisley, UK). C6/36 cells (*Aedes albopictus*; CCLV-RIE-1299) were grown in Schneiders Drosophila media (PAN-Biotech, Aidenbach, Germany) supplemented with 2 mM stable L-glutamine (Capricorn Scientific, Ebsdorfergrund, Germany), 1 mM sodium pyruvate (PAN-Biotech, Aidenbach, Germany), 1X MEM NEAA (PAN-Biotech, Aidenbach, Germany), 100 U/mL penicillin, 100 µg/mL streptomycin (Capricorn Scientific, Ebsdorfergrund, Germany), and 10% FBS (Biowest, Riverside, MO, USA) at 28 °C. THP-1 cells (Homo sapiens, ATCC TIB-202) were cultured in RPMI-1640 Medium (Capricorn Scientific, Ebsdorfergrund, Germany) supplemented with 10% FBS (Capricorn Scientific, Ebsdorfergrund, Germany), 1% penicillin–streptomycin (100 units/mL) (PAN-Biotech GmbH, Aidenbach, Germany), and 0.05 mM 2-Mercaptoethanol (Gibco, Paisley, UK) at 37 °C in 5% CO_2_.

### 2.2. Differentiation of Murine BMDCs and Human DCs

Murine bone marrow-derived dendritic cells (BMDCs) were generated by cultivating primary bone marrow cells from the tibia and femur of 16 different C57BL/6 mice in Iscove’s modified Dulbecco’s medium (IMDM) (Capricorn Scientific, Ebsdorfergrund, Germany) supplemented with 10% FBS, 2 mM L-glutamine, 100 U/mL penicillin, 100 µg/mL streptomycin, and 10% supernatant derived from X63 cells, which contains granulocyte-macrophage colony-stimulating factor (GM-CSF) [50]. The differentiation process was carried out for 9 days, with a medium exchange on days 3 and 5. After differentiation, the cell flasks were rinsed with a culture medium to collect all semi-adherent and non-adherent cells. Successful differentiation was finally confirmed by CD11c expression levels of ≥70%, determined by flow cytometry (Figure S1A).

For THP-1 cells differentiated into dendritic cells (hDCs), all steps were executed in accordance with the mDC differentiation protocol conceptualised by Hölken and Teusch [51]. In short, 1 × 10^6^ THP-1 cells were cultured for 72 h at 37 °C and 5% CO_2_ in 5 mL serum-free RPMI 1640 medium supplemented with 1% penicillin–streptomycin, 10% FBS, 100 ng/mL rhGM-CSF (Gibo, Paisley, UK), 200 ng/mL rhIL-4 (Gibco, Paisley, UK), 20 ng/mL TNF-a (Gibo, Paisley, UK), and 200 ng/mL ionomycin (Biozol, Eching, Germany). Adherent cells were detached with the aid of accutase (Capricorn Scientific, Ebsdorfergrund, Germany). For the control of differentiation, the cells were eventually checked for high CD11c and CD86 expression using flow cytometry (Figure S1B).

### 2.3. Flow Cytometry Assay

Differentiated BMDCs were washed twice with PBS containing 0.5% FBS and blocked with anti-mouse CD16/32 (1:100, clone 93, Thermo Fisher Scientific Inc., Waltham, MA, USA) for 10 min at 4 °C. Subsequently, APC-conjugated anti-mouse CD11c (1:250 dilution, clone N418, Thermo Fisher Scientific Inc., Waltham, MA, USA) was added for 20 min at 4 °C in the dark. Following two washing steps, the stained cells were resuspended in PBS containing 0.5% FBS. The collected hDCs were washed two times, followed by a 10 min blocking step at room temperature, using Human TruStain FcX (BioLegend, San Diego, CA, USA). In the following, an Alexa Fluor^®^ 488 anti-human CD11c Antibody (BioLegend, San Diego, CA, USA) and a PE anti-human CD86 Antibody (BioLegend, San Diego, CA, USA) were applied for 20 min at 4 °C in the dark. For viability staining, the 7-AAD Viability Staining Solution (BioLegend, San Diego, CA, USA) was used. The final examination of both cell types was performed using the Attune NxT flow cytometer (Thermo Fisher Scientific Inc., Waltham, MA, USA), followed by data analysis using the FlowJo software (Version 10, Tree Star, Ashland, OR, USA).

### 2.4. Viruses Culture and Infection of Mammalian Cells

BATV [52], BUNV [53], lNRIV, and BAYAV [49] were propagated on BHK-21 and C6/36 cells in alternating steps. The infection of mammalian cells was conducted with a virus harvested from infected C6/36 cells. T25 cell culture flasks were used for all virus infection experiments using murine BMDCs and hDCs, while T75 flasks were used for the infection of SFT-R cells. The total cell count of each replicate (n = 3 per virus and cell type) was split in half, enabling the use of one half for mock infection and one half for viral infection with an MOI of 0.1 in the FBS-free culture medium. After 1 h of incubation at 37 °C and 5% CO_2_, the medium was exchanged for the respective culture medium with 10% FBS. After 24 h the infection cells were harvested, washed with 1× PBS and forwarded to RNA extraction. Additionally, the supernatant was collected for evaluation of the viral titer.

### 2.5. Viral Growth

The viral titer was determined using the median tissue culture infective dose (TCID50) assay. Briefly, BHK-21 cells were grown in T25 cell culture flasks until confluency was reached. Following a Trypsin-EDTA based detachment (0.05%) (Capricorn Scientific, Ebsdorfergrund, Germany), a cell suspension with 1 × 10^5^ cells/mL in MEM-E with 10% FBS, 2 mM stable L-glutamine and 1% penicillin–streptomycin (100 units/mL) was made. Each well of a 96-well plate was supplemented with 99 µL MEM-E. The wells of the first column were completed by adding 11 µL infection supernatant per well. After performing serial dilutions by transferring 11 µL from each column into the next, a dilution range from 10^−1^ to 10^−10^ in eight replicates was reached. Subsequently, 100 µL of the prepared cell suspension was added per well. After 5 days of incubation at 37 °C and 5% CO_2_, the cytopathic effects were assessed, and the titer was calculated in accordance with Reed and Muench [54].

### 2.6. RNA Extraction

The RNA extraction from OBV-infected cells for qPCR (BMDCs, hDCs, SFT-R) and RNA sequencing (BMDCs) was performed using the same protocol. In short, harvested cells were resuspended in QIAzol Lysis Reagent (Qiagen, Hilden, Germany), following the manufacturer’s protocol for lysis and homogenisation using the TissueLyser II (Qiagen, Hilden, Germany) until the aqueous phase was obtained. The aqueous phase was then further processed with the Monarch RNA Cleanup KIT (10 µg) (New England Biolabs, Ipswich, MA, USA) following the manufacturer’s manual. Residual DNA was removed using DNase I, RNase-free (1 U/µL) (Thermo Fisher Scientific Inc., Waltham, MA, USA).

### 2.7. Sequencing Analysis of Differential Expression and GO Term Enrichment

All BMDC-RNA samples underwent quality control using a High Sensitivity RNA ScreenTape assay on a TapeStation system (Agilent, Santa Clara, USA). Libraries were prepared according to standard polyA-enrichment protocols using the NEBNext Ultra II Directional RNA Library Prep Kit for Illumina (New England Biolabs, Ipswich, MA, USA). In the next step, these libraries were sequenced on an Illumina NextSeq2000 for 2 × 100 bp, targeting 50 million reads. The processing of the raw sequence files was conducted in Python (version 3.7.12) and in the R programming environment (version 4.0.2, www.r-project.org). The quality of the raw sequenced files was evaluated using the FASTQC tool (version 0.12.1) [55]. All files showed sufficient quality for downstream analyses. Reads were mapped to the *Mus musculus* reference genome (GRCm39, obtained from https://www.ensembl.org/Mus_musculus (accessed on: 31 July 2024)) using STAR (version 2.5) [56] with the quantMode GeneCounts parameter, thereby generating gene-level count data. Differential expression analysis, performed using the DESeq2 package (version 1.30.1) [57], was conducted on the generated count data. Prior to conducting the differential expression assessment, any genes with an overall low count of less than 10 reads per gene were removed. *p*-values were adjusted to control the false discovery rate (FDR) using the Benjamini–Hochberg method [58]. Pairwise comparisons were made between each infection group and the mock group. The Gene Ontology (GO) enrichment analysis of each gene set was performed using the R package clusterProfiler (version 4.11.1) [59] in the R programming environment (version 4.4.0) after the exclusion of genes that were not present in the sequencing data of all four infections.

### 2.8. qRT-PCR, qPCR and Gene Expression Calculation

In this study, both quantitative RT-PCR (qRT-PCR) and quantitative PCR (qPCR) were performed. Isolated BMDC and hDC RNA were subjected to first-strand cDNA synthesis using the Luna Script RT SuperMix (New England Biolabs, Ipswich, MA, USA). To assess the gene expression levels TaqMan^®^ Gene Expression Assays (Thermo Fisher Scientific Inc., Waltham, MA, USA) for *Actb* (Hs99999903_m1, Mm02619580_g1), *Ifna6* (Hs00819627_s1, Mm01703458_s1), *Ifnb1* (Hs01077958_s1, Mm00439552_s1), *Isg15* (Hs00192713_m1, Mm01705338_s1), *Isg20* (Hs00158122_m1, Mm00469585_m1), *Cxcl9* (Hs00171065_m1, Mm00434946_m1), *Cxcl11* (Hs00171138_m1, Mm00444662_m1), and *Cfh* (Hs00962373_m1, Mm01299248_m1) were used. The master mix composition and thermal program settings were prepared in accordance with the manufacturer’s manual. Regarding the SFT-R RNA, the SYBR Green-based Luna Universal One-Step RT-qPCR Kit (New England Biolabs, Ipswich, MA, USA) was used in combination with gene-specific primers, as listed in Table A1. The thermal cycling program consisted of an initial reverse transcription step at 55 °C for 10 min, followed by an initial denaturation at 95 °C for 1 min. The amplification included 40 cycles of 10 s denaturation at 95 °C and 30 s extension at 60 °C. Subsequently, a melting curve analysis was run, ranging from 60 °C to 95 °C, with increments of 0.2 °C per second. In the case of the *Cxcl11* qRT-PCR, the extension temperature was changed to 54 °C. The qRT-PCR, as well as the qPCR, were performed on a LightCycler^®^ 96 Real-Time PCR System (Roche, Basel, Switzerland). Finally, the 2^−ΔΔCt^ method after Livak and Schmittgen [60] was used to calculate qPCR-based relative fold changes in the gene expression. Non-detects were set to 40 to enable calculations. Cq values were normalised to *Actb* as a reference gene, and the fold change was calculated relative to the mock-infected samples. For a more intuitive data presentation, all fold changes were log2-transformed.

### 2.9. Data Visualization

Data visualisation was performed utilising the packages ggplot2 (version 3.5.1) [61], EnhancedVolcano (version 1.21.0) [62], pheatmap (version 1.0.12) [63], and ggpubr (version 0.6.0) [64] in the R programming environment (version 4.4.0).

### 2.10. Statistical Analyses

Apart from the processing of the RNA sequencing, all statistical analyses were performed using the R programming environment (version 4.4.0) with the R package Rstatix (version 0.7.2) [65]. Normality was assessed using the Shapiro–Wilk test, while the homogeneity of variance was evaluated using Levene’s test. For parametric analyses of multiple groups, a one-way ANOVA followed by Tukey’s post-hoc test for multiple pairwise comparisons was utilised. In the case of non-parametric data, the Kruskal–Wallis test, followed by Dunn’s test, was used for the statistical analysis. Results with *p* ≤ 0.05 were considered statistically significant. The significance levels were visualised as follows: *p* > 0.05 (ns), *p* ≤ 0.05 (*), *p* ≤ 0.01 (**), and *p* ≤ 0.001 (***).

## 3. Results

### 3.1. Transcriptomic Analysis of OBV-Infected Murine BMDCs

**Figure 1:**
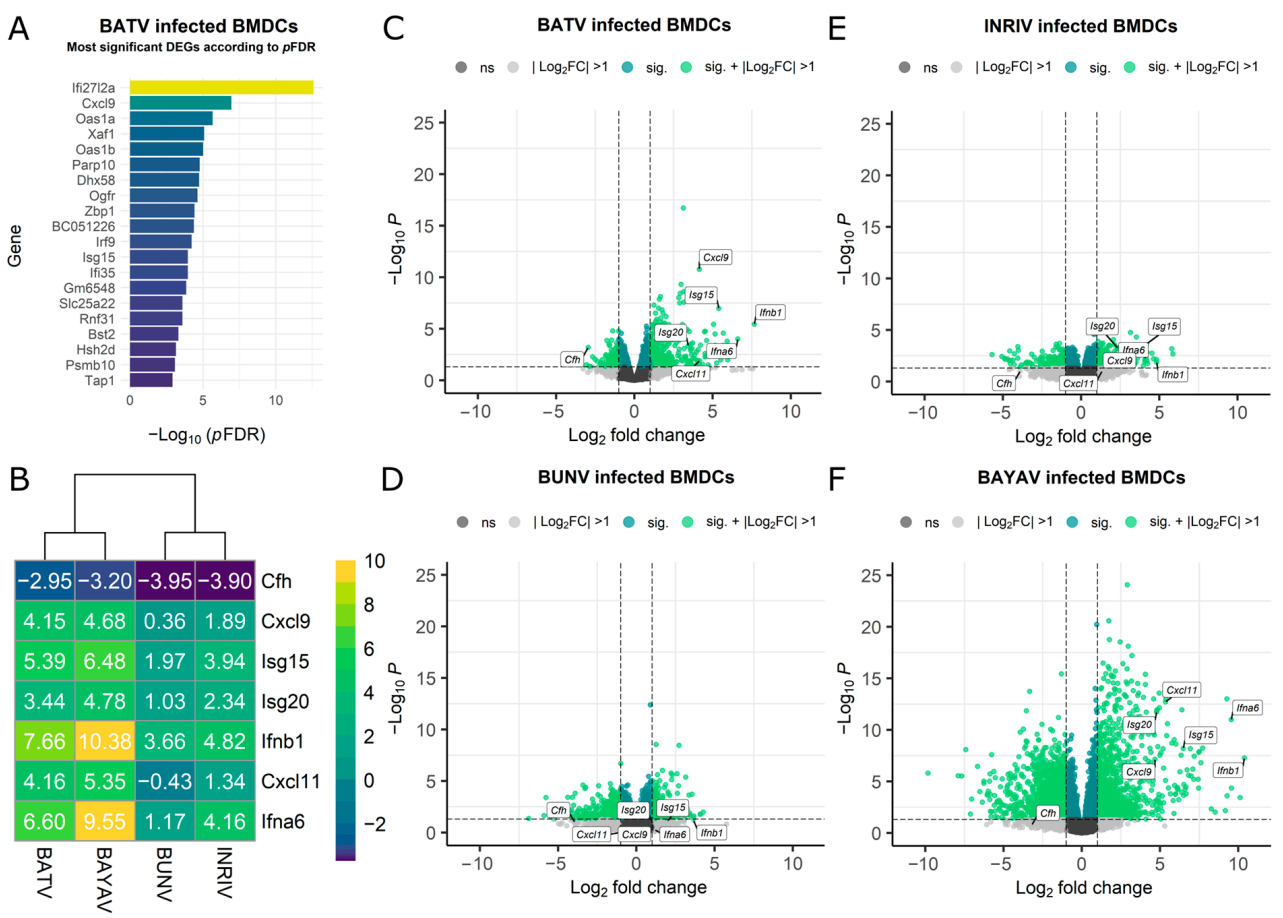
RNA-seq data representing the gene expression of Orthobunyavirus-infected murine BMDCs. (**A**) Bar diagram displaying the top 20 differentially expressed genes following an OBV infection, ranked by adjusted *p*-value (*p*FDR). (**B**) Heatmap showing the log2 fold change (log2FC) of genes representative of antiviral mechanisms and innate immune activation. (**C**–**F**) Volcano plot displaying the log2FC (x-axis) versus −log10 (*p*-value) (y-axis). The threshold in the volcano plot is set to −log10 (*p*-value) = 0.05 (dark green) and absolute log2FC >1 (light green). Not significantly affected genes or values not passing the foldchange threshold are displayed in grey. Selected antiviral and innate immunity markers are labelled for clarity.

RNA sequencing data from BMDCs infected with BATV, BUNV, and their reassortants (BAYAV and lNRIV [49]) were used to explore the cellular transcriptome for significant alterations. The analysis revealed a substantial number of differentially expressed genes (DEGs) after the BATV infection throughout all DEG definitions (Table 1). In contrast, lNRIV infections led to the lowest DEG count, while BUNV, and especially BAYAV, induced a noticeably greater change (Table 1). Interestingly, when ranked by significance, genes related to an early immune response (e.g., *Cxcl9*, *Rnf31*, and *Ifi35*) and antiviral immune response (e.g., Oas-family members, *Isg15*, and *Xaf1*) dominated the top DEGs after BATV infection (Figure 1A). This high abundance of immune response genes among the top-ranked genes was not observed in BUNV infection and appeared to a markedly lower extent after BAYAV and lNRIV infection (Figure S2).

Next, we decided to perform Gene Set Enrichment Analysis (GSEA), applying a less stringent DEG definition (*p*-value < 0.05 and a |log2FC| > 1), as GSEA benefits from a broader dataset to ensure sufficient gene inclusion for pathway analysis.

The GSEA confirmed that especially genes associated with antiviral mechanisms and innate immune activation showed high log2FC values and significance after BATV infection (Figure S3A, Table S1). lNRIV-infected cells also prioritised antiviral mechanisms and innate immune responses, while BAYAV-infected cells exhibited a partial shift in focus toward cell division and proliferation. In contrast, BUNV-infected cells showed mainly changes in genes associated with cell division (Figure S3B–D, Table S1). This change in the involvement of early antiviral genes among the OBV infections was well reflected in the clear expression shift of the seven selected immune response genes chosen as representatives for an antiviral immune response (Figure 1C–F). This selection was based on a mix of top-ranked genes and genes known to play major roles in early antiviral processes (*Cfh*, *Ifna6*, *Ifnb1*, *Isg15*, *Isg20*, *Cxcl9*, *Cxcl11*) (Figure 1B).

**Figure 2:**
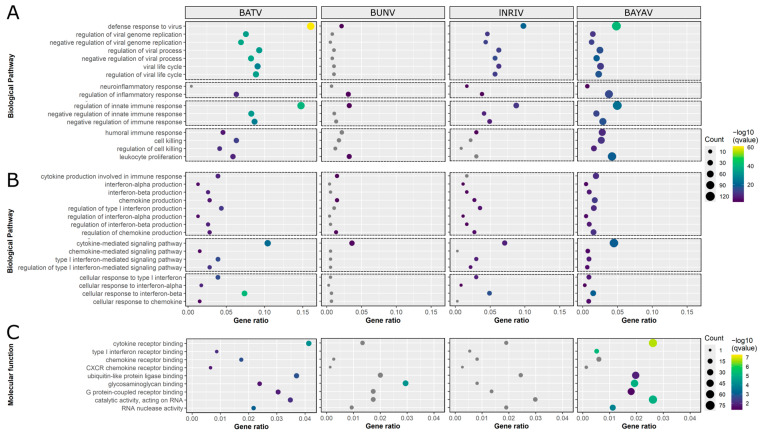
Dot plots of RNA-seq data-based Gene Ontology (GO) terms associated with antiviral innate immunity. GO terms are visualised on the y-axis, and gene ratio on the x-axis. The size of the dots corresponds to the number of differentially expressed genes (DEGs) associated with each GO term, while the colour depicts the FDR-corrected *p*-value (qvalue). (**A**,**B**) Overview of Biological Pathway GO terms related to (**A**) a general early antiviral immune response and (**B**) cytokine-specific terms. (**C**) Selected Molecular Function GO terms representative of antiviral mechanisms and innate immune activation.

Due to the substantial differences in the expression of immune-response-related genes, we followed up with GSEA focused on GO terms associated with an early immune response that primarily contained one or more of the seven selected genes (Figure S4). Although similar patterns were observable, the extent of pathway involvement differed in significance, gene ratio, and gene count (Figure 2A, Table S2). While BATV infections resulted in the most significant changes in virus-related GO terms, BUNV induced very few alterations. Interestingly, the concentrations of DEGs associated with neuroinflammatory response were only affected by the reassortants. GO terms associated with an immune response regulation were, as before, most notably impacted following BATV infections. Concerning GO terms of the humoral and general cellular immune response, BUNV and lNRIV infections only significantly affected the terms humoral immune response (lNRIV), lymphocyte-mediated immunity (BUNV), and leukocyte proliferation (BUNV), while BATV and BAYAV induced all terms.

When focusing on GO terms specific for cytokine production, signalling, and response, BATV infections induced the greatest change, followed by lNRIV, BAYAV, and, lastly, BUNV (Figure 2B, Table S2). Notably, the only cytokine-related aspect where BUNV had an impact was in DEGs associated with chemokine production.

Looking into relevant Molecular Function (MF) GO terms, it was further confirmed that a BATV infection altered initial immune-response-related GO terms (Figure 2C, Table S3). However, it was BAYAV infections, rather than NRIV, that most closely resembled the response to BATV infection. Noteworthy, BUNV infections led only to significant changes in the GO term glycosaminoglycan (GAG) binding and lNRIV to no changes at all.

In conclusion, it became evident that BATV has a pronounced effect on the expression of genes related to an initial immune response, in stark contrast to the minimal impact observed with BUNV infection. The reassortment events, however, resulted in distinct compromises between these opposing effects, with additional effects unique to reassortants, like the activation of a neuroinflammatory response or the loss of significant GAG binding.

**Figure 3:**
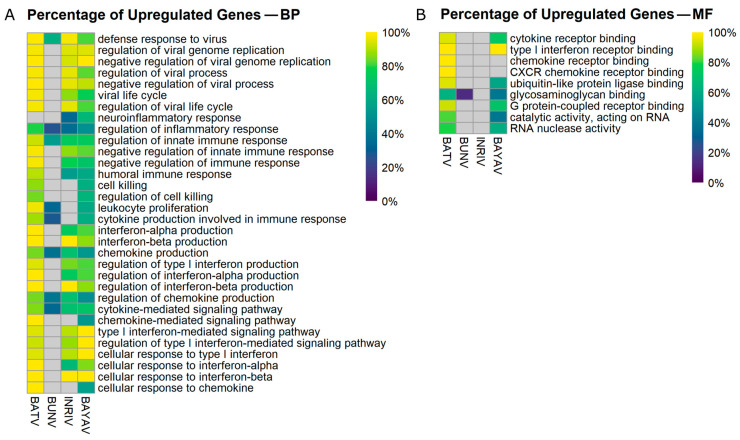
Heat map displaying the percentage of upregulated DEGs in selected Gene Ontology (GO) terms. The heatmap illustrates the proportional upregulation of differentially expressed genes (DEGs) across selected Biological Pathway (BP) GO terms (**A**) and Molecular Function (MF) GO terms (**B**).

An additional finding was that BATV infection predominantly upregulated DEGs in the selected BP GO terms, while BUNV infections tended to promote downregulated DEGs (Figure 3A, Table S4).

These patterns became shuffled up in the reassortants. Concerning virus-related GO terms, both reassortants mirrored the BATV infection. However, for the inflammatory GO terms as well as for the immune response regulation associated GO terms, a distinct increase in downregulated DEGs was observable for the reassortants. A predominance of upregulated DEGs was present for especially interferon-beta-related GO terms in lNRIV infections. BAYAV infections promoted upregulated DEGs, especially in the context of IFN-I signalling pathways, regulation, and response (Figure 3A, Table S4). In a similar manner, the BATV infection mainly led to upregulated DEGs in the selected MF GO terms, while BAYAV (and BUNV) infected cells produced higher amounts of downregulated DEGs (Figure 3B, Table S4).

Overall, this indicates that a BATV infection emphasises high immune gene upregulation, while infections with the reassortants introduced an increased number of downregulated genes into the DEG pool.

### 3.2. Interspecies Differences of Innate Immunity Markers After OBV Infection

**Figure 4:**
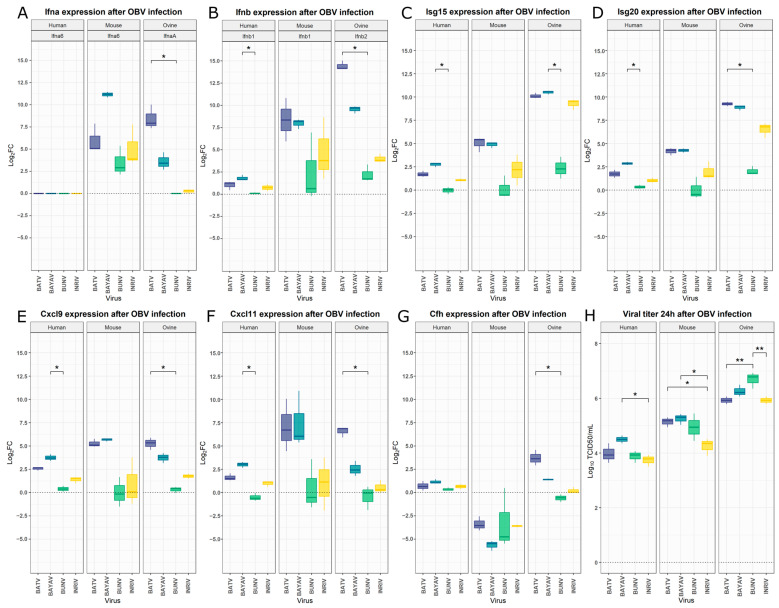
Differential expression levels after OBV infection in human, mouse, and ovine cells. Log2 fold changes (log2FC) between uninfected and infected samples were determined by qPCR using the 2^−ΔΔCt^ method. (**A**) Expression of *Ifna6* in murine BMDCs and THP-1-derived DCs, and *IfnaA* in SFT-R cells after OBV infection. (**B**) *Ifnb1* expression in murine BMDCs and THP-1-derived DCs, and *Ifnb-2* in SFT-R cells. Expression levels of *Isg15* (**C**), *Isg20* (**D**), *Cfh* (**E**), *Cxcl10* (**F**), and *Cxcl11* (**G**) in OBV-infected cells. (**H**) Viral titer of BATV, BAYAV, BUNV, and lNRIV 24 h post-infection. The significance of log2FC in gene expression was evaluated using the non-parametric Kruskal–Wallis test, followed by Dunn’s post-hoc test for multiple comparisons. Viral titers were analysed using one-way ANOVA followed by Tukey’s post-hoc test for pairwise comparisons. Statistical significance is indicated as follows: *p* ≤ 0.05 (*) and *p* ≤ 0.01 (**).

To compare our findings from murine BMDCs with other relevant host species of OBVs, namely, humans and sheep, we decided to extend our study by examining the expression of seven selected genes in human (hDCs) and ovine (SFT-R) cells. The analysed genes were identical, with the exception that, for the ovine samples, *IfnaA* and *Ifnb2* were selected instead of *Ifna6* and *Ifnb1* due to the absence of the latter in the ovine genome. We also infected a new group of murine BMDCs to confirm the results of the RNA sequencing and to ensure comparability between the species. For all seven genes, the log2FC obtained during RNA sequencing was within the standard deviation of the log2FC evaluated by qPCR (Figure 4A–G; Table S5). A consistent pattern was observed across all tested genes and species, with BATV and BAYAV infections clustering together alongside BUNV and lNRIV infections. Therefore, BATV and BAYAV induced stronger upregulations in all species for all genes, except for *Cfh* expression in mice. Ovine cells reacted with especially strong upregulation of *Isg15*, *Isg20,* and *Ifnb-2* after BATV, lNRIV, or BAYAV infection. The hDCs showed, in all cases, the weakest response, with *Ifna6* even being undetectable (Figure 4A–G; Table S5). A Kruskal–Wallis test of the hDCs data revealed significant differences in log2FC between the viral infection for all genes but *Ifna6* and *Cfh* (Table S5). In all cases, these differences were based on a significantly higher Log2FC after BAYAV infection compared to BUNV infection (Figure 4A–G, Table S5). Multiple comparison analysis did not yield significant changes in murine BMDCs (Figure 4A–G; Table S5). However, in the case of the infected ovine cells, a virus-specific significant difference in gene expression was revealed for all genes (Figure 4A–G; Table S5). For most genes, this difference is based on a significantly higher gene expression induction after a BATV infection than after a BUNV infection (Figure 4A,B,D–G; Table S5). Yet, in the case of *Isg15*, it was the significantly higher expression in BAYAV-infected cells compared to BUNV-infected cells (Figure 4C; Table S5).

In summary, these data show that a reassortant virus can have significantly different effects on the expression of immune-response-related genes for human and ovine immune cells, but not murine. During these infections, BATV and BAYAV, as well as lNRIV and BUNV, instigated similar changes, with BATV and BAYAV exhibiting greater immunogenicity.

Ultimately, we also investigated the viral titer achieved by these viruses within the first 24 h in each cell type. With mean log10 TCID50/mL of 5.93–6.69, the highest titers were reached in the ovine cells, followed by a substantial drop in murine BMDCs (log10 TCID50/mL 4.25–5.14) and then hDCs (log10 TCID50/mL 3.74–4.50). In the hDCs, a significantly higher viral titer was measurable in BAYAV-infected cells compared to lNRIV-infected cells. The same significant difference was observed in the murine BMDCs, accompanied by significantly higher BATV titers compared to the lNRIV titer. Interestingly, the viral titer in the infected ovine cells was highest for BUNV, which significantly exceeded lNRIV and BATV (Figure 4H, Table S6). Cumulatively, this highlights that closely related and reassorted OBVs can grow significantly differently within and between species.

## 4. Discussion

Our study provides the first transcriptome-based insights into the early response of mammalian immune cells upon infection with the OBVs BATV and BUNV, as well as the changes that reassortment can induce.

It was shown that murine BMDCs, after infection with BATV, strongly induce genes associated with the activation of an innate immune response and antiviral mechanisms. Genes involved in the IFN-I response were especially highly upregulated, which is expected since bunyaviruses replicate in the cytosol and are thus sensitive to cytosolic innate immune mechanisms [39,40], as has been proven by the IFN sensitivity shown for multiple OBVs [66,67]. However, numerous OBVs have developed mechanisms to counteract this antiviral response. This is evidenced by the fact that the NSs protein encoded by the S-segment of various OBVs acts as an IFN induction antagonist. Examples of such viruses are BUNV [32,37,38,43], LACV [68], SBV [69], Rift Valley Fever virus (RVFV) [70], CVV and Kairi virus (KRIV) [71]. Consequently, our transcriptome data for BATV suggest that it exhibits a less effective blocking of the early immune response. It appears compelling to claim that this contrast is most likely associated with differences in the NSs-protein. Yet, both reassortants also show these signs of a reduced ability to counteract the immune response, even though one shares the S-segment with BUNV (lNRIV: BUNV L, BATV M, BUNV S) and the other with BATV (BAYAV: BUNV L, BATV M, BATV S) [49]. This indicates that the resulting immune response phenotype after reassortment is not solely influenced by the NSs inheritance. Since both reassortants share the M-segment with the BATV, a substantial part of the immune system stimulation might be attributed to the inheritance of M-segment-encoded glycoproteins or the NSm protein. Unfortunately, there is less information about the NSm of OBVs than the NSs [72]. However, Ishihara, et al. [73] revealed the influence of the NSm protein on the pathogenicity of Akabane virus. In conclusion, our results suggest that both the NSs and NSm proteins of BATV may be less effective in counteracting the early immune response, at least in the murine model.

The potential for a strong cytokine reaction inherited from BATV might result in reassortants with an increased pathogenicity. Research has shown that high cytokine levels—often characterised as a cytokine storm—define the severity of Severe Fever with thrombocytopenia syndrome virus (SFTSV) or Lassa fever (LASV) infections [74,75]. Therefore, this provides potential groundwork for explaining the pathology of the natural reassortant NRIV.

Although both reassortants display the same signs of reduced immune evasion as BATV, it should be noted that a higher proportion of downregulated DEGs in each GO term was observed, especially for BAYAV. This could indicate an increased level of viral immune suppression. For other bunyaviruses like Crimean-Congo haemorrhagic fever virus (CCHFV), a deubiquitinase function of the L-segments was discovered [39,76,77]. A function that BUNV potentially also possesses and thus would explain why the BAYAV reassortant carrying the BUNV L-Segment shows a less significant early immune system induction than BATV, despite them sharing both “virulence” segments.

An additional fascinating aspect of BAYAV is the noticeably higher DEG count. A significant portion of these additional DEGs is involved in processes of cell division and proliferation. It has been thoroughly proven that viruses can interfere with cellular proliferation, either to their advantage or detriment [78]. In line with this, there is also evidence that BUNV infections manipulate the cell cycle [79]. Consequently, it seems plausible that the high number of DEGs following a BAYAV infection could result from increased immunogenicity combined with manipulation of the host cell proliferation. However, this hypothesis would only be valid if the function affecting the cell proliferation is located on the L-segment, as BUNV and BAYAV only share this segment. The presence of a deubiquitinase on the BUNV L-segment would support this hypothesis, as viral deubiquitinases are known to interfere with cellular processes, including proliferation [80].

The previously mentioned predominance of downregulated DEGs in the reassortants was present across all GO terms. Especially genes associated with chemokines were affected by this downregulation after BUNV but also BAYAV and lNRIV infections. This is further supported by the negative or missing influence of BAYAV and lNRIV on leukocyte proliferation since chemokines especially shape leukocyte activity and recruitment [81]. Thus, reassortment might lead to viral protein combinations with novel effects on the immune response. Notably, leukocyte levels are associated with bleeding during intracerebral haemorrhage [82] and the severity of CCHFV [83]. Intracerebral haemorrhage and the fact that reassortant lNRIV and BAYAV significantly influenced the amounts of genes coupled to a neuroinflammatory response might also lead to a unique pathology of reassortant bunyaviruses.

Furthermore, it was shown that BATV and BAYAV show a significant amount of DEGs related to cell killing and its regulation, while BUNV and lNRIV do not. This supports the assumption that the BATV NSm is pro-apoptotic, as seen in the case of LACV or SBV [84,85], in contrast to the anti-apoptotic function of the NSm of BUNV [37], again supporting the assumption that BUNV S-segment mediates stronger immune evasion than BATV S-segments.

Through the analysis of the seven selected genes, it could be shown that the listed differences are potentially transferable into the ovine and human systems. These systems are worth investigating due to sheep being part of the livestock industry significantly affected by OBVs [1,86,87] and humans being recurrently exposed to the zoonotic potential of OBVs [16,25]. The qPCR data support the transcriptome data by showing that a reassortant can exhibit significantly altered expression of immune response genes when compared to the parental virus. In this case, there is an especially significantly heightened gene expression of IFN-I in the BAYAV reassortant. This inability of BATV and BAYAV to effectively counteract the immune response could explain why there are still very few symptomatic reports, although the seroprevalence suggests substantial circulations of BATV [27,28], and also why a reassortant with a similar genetic composition to BAYAV was not detected in nature. In addition, the similarity in gene expression between the murine, ovine, and human cell systems allows the assumption that the innate immune response in the different hosts might be alike and that the transcriptomic data of the infected murine cells may be applicable to human and ovine systems. However, ovine cells strikingly increased their interferon-stimulated gene (Isg) expression after BATV, BAYAV, and lNRIV infection. *Isg15* is a ubiquitin-like protein that is particularly strongly induced in viral infections [88,89]. The ovine immune system is no exception since a major hub role against viral stimulus was reported for *Isg15* and, to a lesser extent, *Isg20* [88]. Sheep are particularly studied due to their significant upregulation during early pregnancy. Interestingly, studies have shown that there is an association between decreased Isg levels and pregnancy [90,91,92]. The stark contrast in *Isg* regulation between BUNV and the other three OBVs in this study might be one reason for the association of BUNV with reproductive disorders. Furthermore, this could imply that natural reassortants similar to lNRIV should raise fewer abortion phenotypes. The vulnerability of the ovine cells to BUNV is also mirrored in the significantly fast viral growth of BUNV. Generally, it can be concluded that the ovine cells are the most sensitive regarding virus replication. This is in line with ruminants being most commonly associated with disease after OBV infection [2].

Lastly, the reassortant lNRIV stood out with significantly low viral titers in all tested species. Such a lower growth rate could help to circumvent the immune surveillance [93]. Yet, as other studies show, this is not the case for all investigated host species [47,49,94].

It should be noted that, despite novel insights into OBV–host interactions, several limitations of this study must be taken into account. First, our in vitro setting does not fully mimic the in vivo conditions, where immune and tissue interactions could influence viral dynamics. This includes the use of immortalised cell lines and associated drawbacks [95], as well as the use of a subset of viral strains, which may not fully reflect natural populations [49,96]. In line with this, one needs to consider that our choice of immune cells reflects their known roles in the early stages of arbo-bunyavirus infection and replication [97]. These cells are critical in shaping the immune response and facilitating viral spread, as shown for RVFV and Uukuniemi virus (UUKV) [98,99]. By focusing on immune cells, our study strengthens the current model of arbo-bunyavirus–host interactions but does not address alternative infection strategies, such as epithelial cells, which are common initial targets for many viruses but whose role in arbo-bunyavirus infection remains unclear [97]. Moreover, this study was limited to a one dpi window, capturing early replication but missing later stages, including the adaptive immune response. Thus, future studies are essential and should cover animal models, a broader range of viral isolates, and longer-term studies to further explore the mechanisms and suggestions given in this study.

Nonetheless, this does not diminish the pivotal aspect of this paper, which is to provide a basis and direction for further research concerning OBVs, especially BATV and NRIV. This is research that is urgently needed at the current time, where arbovirus-caused epidemics among humans and domestic animals are on the rise [4,100].

## 5. Conclusions

In summary, we provide comprehensive insights into how the OBVs BATV and BUNV are initially recognised by the immune system and how reassortment affects this response, revealing that BATV seems to be drastically more immunogenic than BUNV, which, in turn, remains largely undetected. Further, we demonstrate that reassortants of both viruses show distinctly changed phenotypes with regard to the immune response, which appears to be compromises between the responses induced by the parental viruses while also exhibiting unique adaptations specific to the reassortants. Additionally, our results suggest that all these observations are present across species boundaries, thus suggesting that reassortment-based changes in the initial immune response could be involved in the sudden occurrence of unexpected pathological patterns in novel OBVs.

## Figures and Tables

**Table 1:** Count of differentially expressed genes (DEGs) after viral infection according to distinct DEG definitions.

Virus	*p*-Value < 0.05	*p*-Value < 0.05 |log2 Fold Change| > 1	*p*FDR < 0.05	*p*FDR < 0.05 |log2 Fold Change| > 1
BATV	1837	550	158	122
BUNV	2983	879	179	86
lNRIV	1372	474	1	0
BAYAV	6179	2742	4465	2234

## Data Availability

Sequencing data were submitted to the sequence read archive (NCBI, project-ID PRJNA1179687). The processed differential gene expression files are available in the Supplementary Materials (Tables S7–S10).

## Supplementary Materials

The following supporting information can be downloaded at: https://www.mdpi.com/article/10.3390/v16121833/s1, Figure S1: Exemplary representation of the differentiation control process and gating strategy for murine BMDCs and THP-1 derived human DCs; Figure S2: RNA-seq data-based bar diagram displaying the top 20 differentially expressed genes following an OBV infection, ranked by adjusted *p*-value (*p*FDR); Figure S3: RNA-seq data-based dot plots of the Top20 Biological Pathway Gene Ontology (GO) terms after OBV infection; Figure S4: Occurrence of selected representative early immune response genes in Biological Pathway and Molecular Function GO terms; Table S1: Gene Ontology Enrichment Analysis—Top20 induced Pathways; Table S2: Gene Ontology Enrichment Analysis—Biological Pathway; Table S3: Gene Ontology Enrichment Analysis—Molecular Function; Table S4: Gene Ontology Enrichment Analysis—Percentual amount of upregulated DEGs; Table S5: qPCR statistics; Table S6: Viral titer statistics; Table S7: Differential gene expression after BATV infection; Table S8: Differential gene expression after BUNV infection; Table S9: Differential gene expression after BAYAV infection; Table S10: Differential gene expression after NRIV infection.

## Appendix A

**Table A1:** Primer for the SYBR Green-based Luna Universal One-Step RT-qPCR of ovine samples.

Primer Name	Gene	Sequence (5′ to 3′)	Size (bp)	Gene Bank	Reference
OSM_1049	Actb_fwd	GAGGCTCTCTTCCAGCCTTC	98	XM_060405599.1	This study
OSM_1050	Actb_rev	CGTAGAGGTCCTTGCGGATG			
OSM_1041	Ifna_fwd	GTGAGGAAATACTTCCACAGACTCACT	107	EU276064	[101]
OSM_1042	Ifna_rev	TGARGAAGAGAAGGCTCTCATGA			
OSM_1056	Ifnb2_fwd	AACAAAGGCGGAGCTCTGTGG	97	XM_004004400.6	This study
OSM_1057	Ifnb2_rev	GGCATCTGGAAGTCCATCCTGAAC			
OSM_1047	Isg15_fwd	AGGTGAAGATGCTAGGGGGC	85	NM_001009735.1	This study
OSM_1048	Isg15_rev	TGGGCGATGAACTGCTTCAG			
OSM_1045	Isg20_fwd	CAGTAGCTGAGAAAGGGGCAT	99	XM_027957054.3	This study
OSM_1046	Isg20_rev	CTCACAGTCCATGGCTACCAC			
OSM_1037	Cxcl9_fwd	GGAGTTCAAGGAATCCCAGCA	120	XM_004009924.6	[102]
OSM_1038	Cxcl9_rev	ACAAGTAGGGCTTGGAGCAA			
OSM_1082	Cxcl11_fwd	CCCAAGTCAAAGCAAGCAAAAGC	91	XM_027971095.3	This study
OSM_1083	Cxcl11_rev	AGTCACAGTTACACTTGTTCTAGGT			
OSM_1039	Cfh_fwd	CGGTGTCAGGCCTACTATGAACT	71	EU888587	[103]
OSM_1040	Cfh_rev	GGTTCTGACCACTCTCCATTCC			
