# Supplementary figures and images for: Innate Immune Response Against Batai Virus, Bunyamwera Virus, and Their Reassortants
Source: Viruses. 2024 Nov 26;16(12):1833. doi: 10.3390/v16121833 (PMC11680289; doi:10.3390/v16121833)

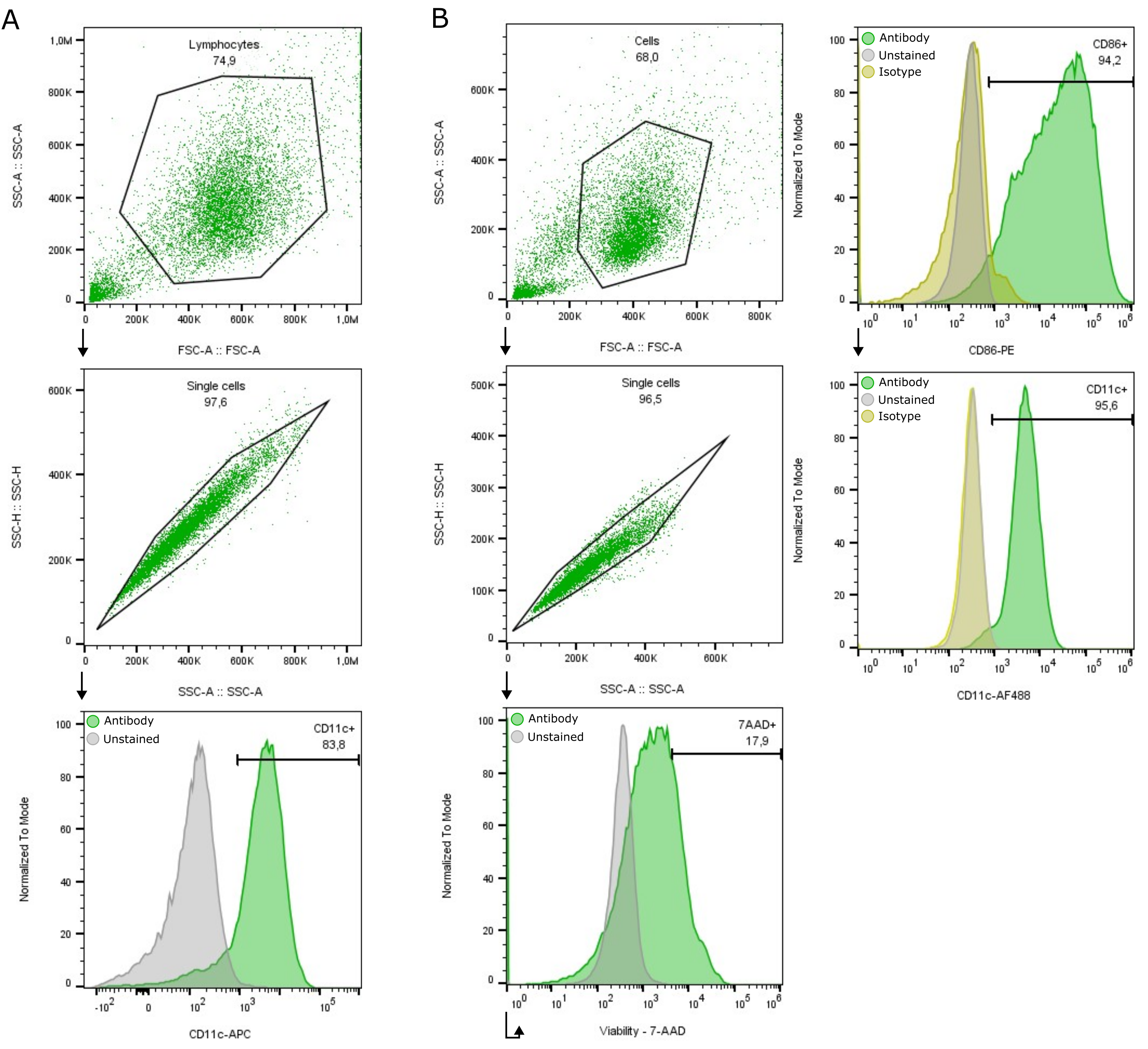

Supplement: Supplementary file 1 [file viruses-16-01833-s001.zip › viruses-3326190-supplementary/Figure_S1.png]

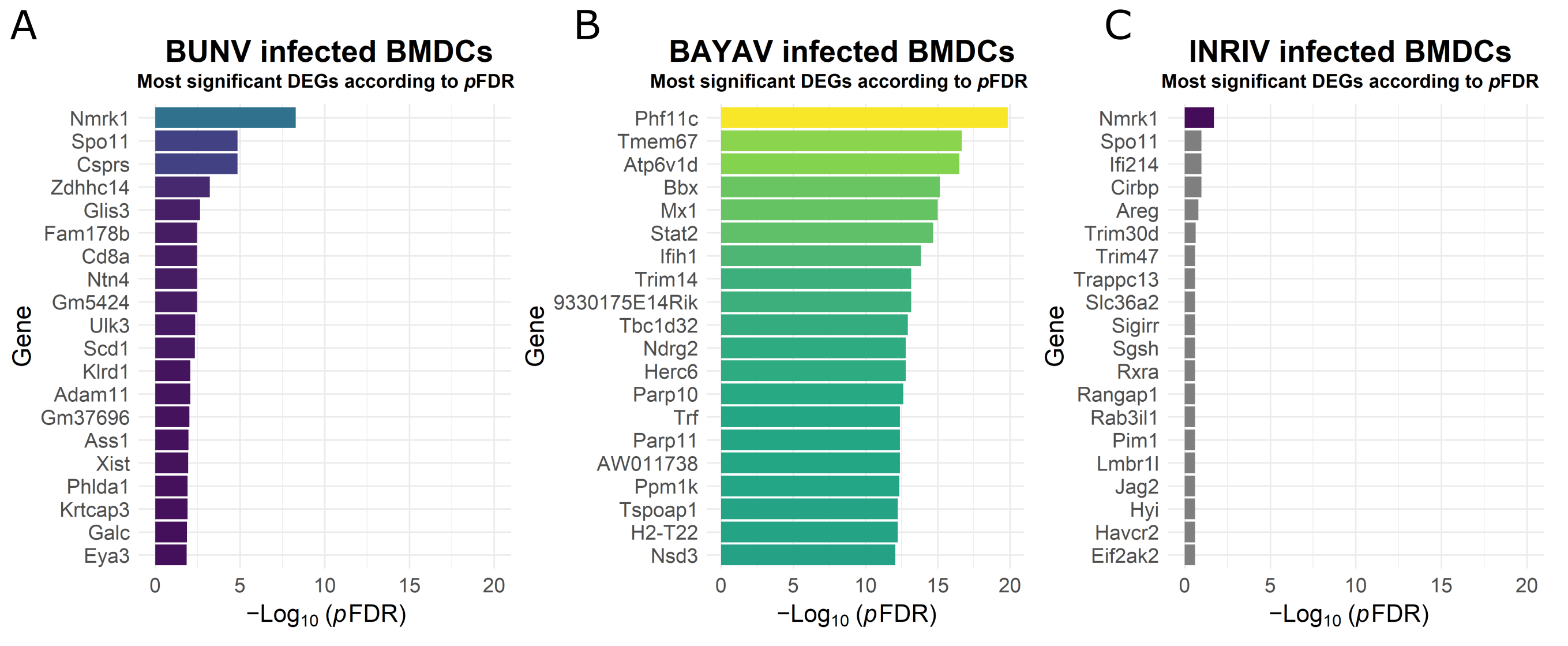

Supplement: Supplementary file 1 [file viruses-16-01833-s001.zip › viruses-3326190-supplementary/Figure_S2.png]

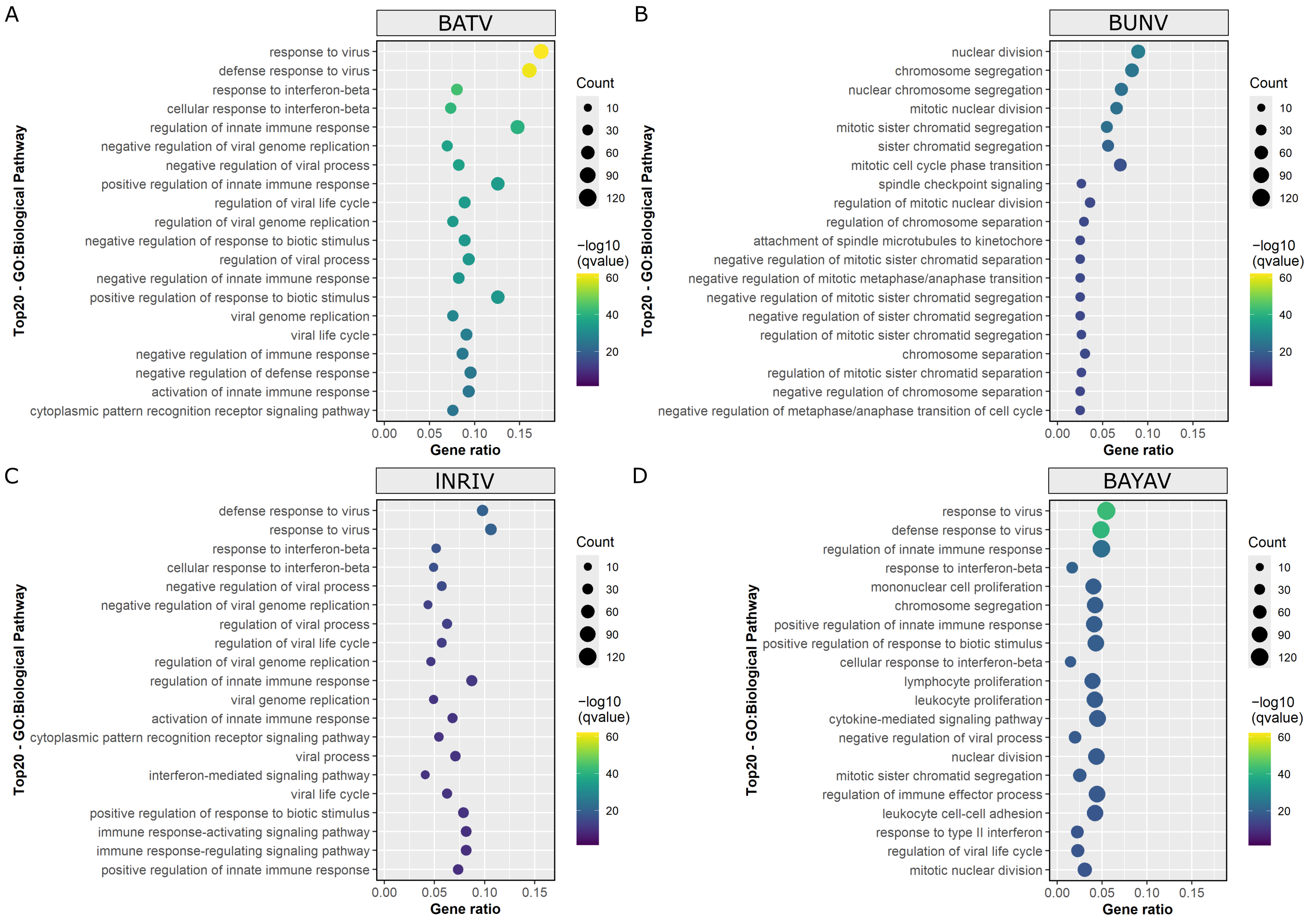

Supplement: Supplementary file 1 [file viruses-16-01833-s001.zip › viruses-3326190-supplementary/Figure_S3.png]

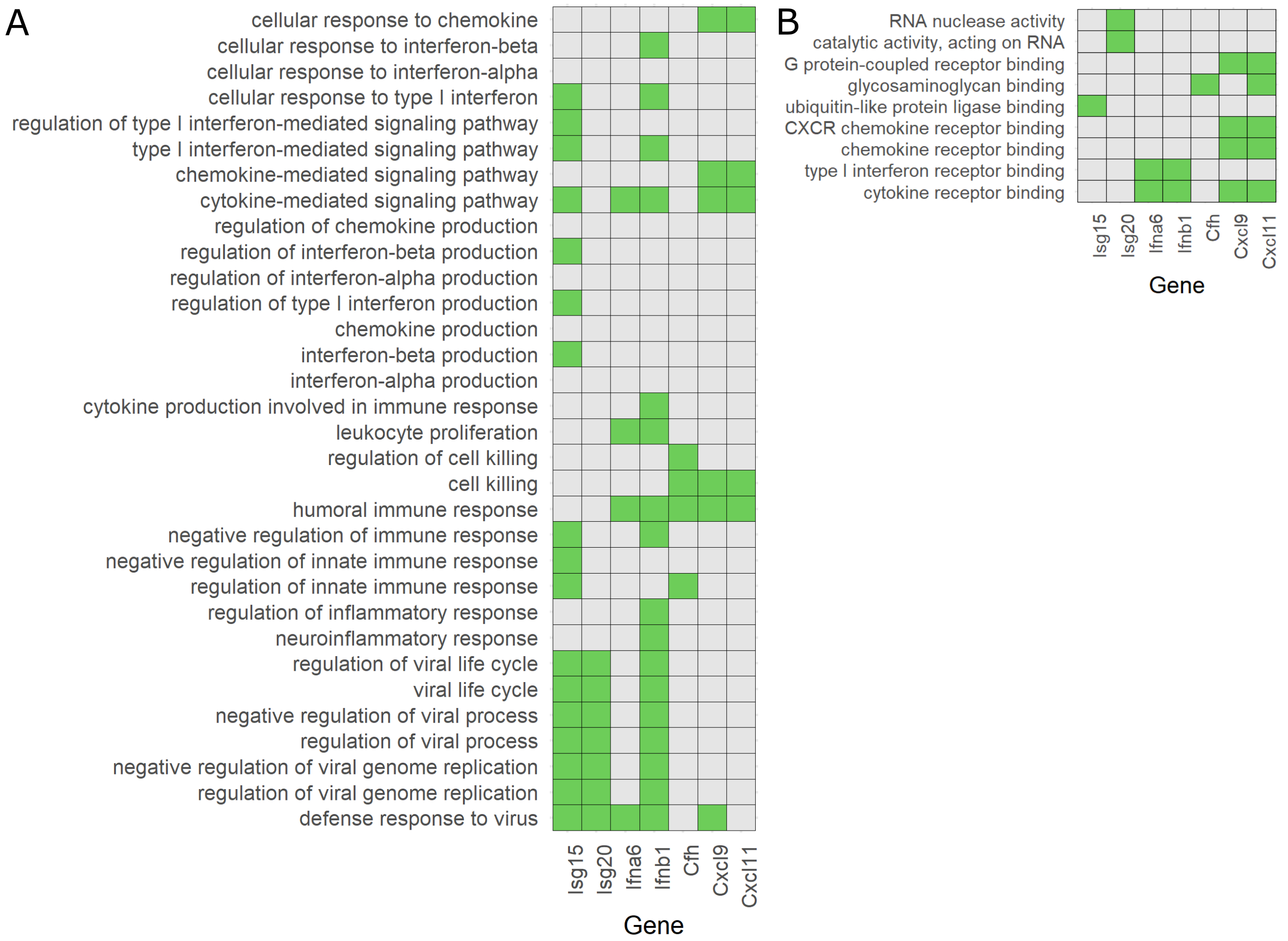

Supplement: Supplementary file 1 [file viruses-16-01833-s001.zip › viruses-3326190-supplementary/Figure_S4.png]
